# Robust low threshold full-color upconversion lasing in rare-earth activated nanocrystal-in-glass microcavity

**DOI:** 10.1038/s41377-024-01671-3

**Published:** 2025-01-02

**Authors:** Zhigang Gao, Lugui Cui, Yushi Chu, Luyue Niu, Lehan Wang, Rui Zhao, Yulong Yang, Xiaofeng Liu, Jing Ren, Guoping Dong

**Affiliations:** 1 College of Physics and Electronic Engineering, Taishan University, 271021 Taian, China; 2 Key Laboratory of In-fiber Integrated Optics of Ministry of Education, College of Physics and Optoelectronic Engineering, Harbin Engineering University, 150001 Harbin, China; 3 School of Materials Science and Engineering, Zhejiang University, Hangzhou, China; 4 State Key Laboratory of Luminescent Materials and Devices, and Guangdong Provincial Key Laboratory of Fiber Laser Materials and Applied Techniques, School of Materials Science and Engineering, South China University of Technology, 510640 Guangzhou, China

**Keywords:** Lasers, LEDs and light sources, Optical materials and structures

## Abstract

Visible light microlasers are essential building blocks for integrated photonics. However, achieving low-threshold (μW), continuous-wave (CW) visible light lasing at room temperature (RT) has been a challenge because of the formidable requirement of population inversion at short wavelengths. Rare-earth (RE)-activated microcavities, featuring high-quality factor (*Q*) and small mode volume of whispering gallery modes, offer a great opportunity for achieving infrared-to-visible upconversion (UC) lasing. Here, we report that batch-produced nano-glass composite (GC) microspheres incorporating RE-doped fluoride nanocrystals show efficient UC emissions. These multi-phase composite microspheres exhibit a high *Q* value (≥10^5^), comparable to that of conventional multi-component glass microspheres. The UC lasing with pure red, green, and blue (RGB) emissions are demonstrated based on a highly efficient tapered fiber-microsphere system. More importantly, the GC microspheres manifest reduced (by 45%) lasing threshold and enhanced (more than four times) slope efficiency. These characteristics, together with excellent long-term stability, suggest a promising solution to achieving highly robust, stand-alone, low-threshold, and versatile UC microlasers.

## Abstract (web summary)

Infrared-to-red, green, and blue upconversion lasing from nano-glass composite microspheres embedded with rare earth ion-doped fluoride nanocrystals is first reported, holding promise for low-threshold microlaser light source applications.

## Graphical abstract

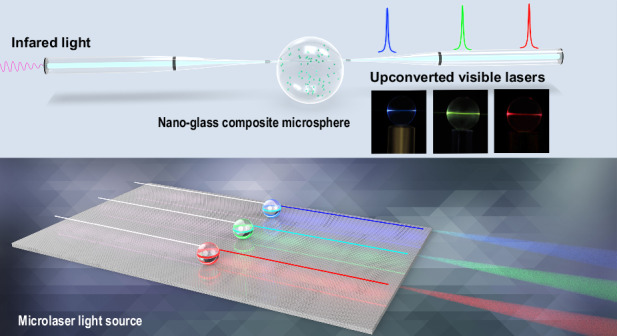

## Introduction

Miniaturized visible light lasers can be readily integrated on chips, deployed in photonic integrated circuits and portable devices. They have been used as essential building blocks for a wide range of applications including full color display, imaging, Li-Fi, optical barcoding and quantum computing^1–8^. Infrared-to-visible light upconversion (UC) emissions appear to be orders of magnitude more efficient than other nonlinear optical processes such as second harmonic generation and optical parametric oscillation. In the recent decade, UC luminescent materials have been widely adopted as the gain media for visible lasing^9,10^. The threshold pump power of UC lasing for rare-earth ion (RE: Tm^3+^, Er^3+^, Ho^3+^, etc.)-activated materials are much lower than the lasers based on quantum dots (QDs) and organic dyes. This is mainly due to the much longer radiative lifetime (microsecond to millisecond) of RE ions as compared with that of QDs and dye molecules (picosecond to nanosecond). The long lifetime facilitates the buildup of population inversion necessary for lasing at short wavelengths^11^. However, as UC emissions occur through the absorption of multiple photons, previous UC lasers made of RE-doped glasses and fibers were only able to operate at very high pump thresholds (several to several hundred mW)^12^. It is essential to reduce the threshold to minimized heat accumulation for on-chip integration of microlasers^13^, which could be promising for bio-sensing applications^7,14^.

Multiphoton UC processes can be significantly enhanced by capitalizing on the Purcell effect, viz., confining and boosting the mode density of optical field around REs^15^. This can be achieved by coating RE-doped fluoride nanocrystals (NCs) on the surfaces of glass or polystyrene microcavities, or using plasmonic nanopillar arrays with a small mode volume (*V*_m_ ≤ 1000 μm^3^). Microspheres supporting resonant whispering gallery modes (WGMs) have proven to be effective in achieving low threshold (μW or even nW) lasing owing to the high pump-to-gain interaction for efficient light coupling to WGMs^15–17^. They are also superior in achieving narrow lasing linewidths (high coherence) than plasmonic microcavities. The latter tends to suffer from line broadening induced by propagation loss^2^. A record low threshold (4 Wcm^-2^) of UC lasing was obtained by improving the surface quality of NCs coated microspheres^6^. The creation of disordered microenvironments for REs was also shown to be important in suppressing phonon-assisted energy back transfer in Yb^3+^/Er^3+^ doped microspheres. As a result, UC lasing with an ultralow threshold (4.7 Wcm^-2^) was obtained^2^. However, the fabrication of the NCs coated microspheres is complicated, and since the coating layer of NCs directly is exposed to environment without protection, the long-term stability of lasing remains as a critical issue especially under harsh environments.

In recent years, there is growing interest in the development of microlasers made of REs-doped multi-component glasses, which offer benefits including: (1) simple preparation and lower cost as compared with crystals, (2) better physical, chemical and long-term stability as compared with polymers^2,13^, and more importantly (3) high doping concentration of REs that permits a large gain per unit length. For example, RE-doped fluoride glasses, such as the well-known (in mol.%) 53ZrF_4_-20BaF_2_-4LaF_3_-3AlF_3_-20NaF (ZBLAN), have been the most often explored gain media. This fluoride possesses a much lower vibrational energy than conventional silica glass. The low phonon energy leads to a reduced multi-phonon relaxation rate, which contributes to prolonged lifetimes of excited states and increased efficiency of UC emissions. In an early report, REs-doped fluoride glass microlaser exhibited a relatively large threshold of 20 mW due to the poor coupling efficiency^18^. Through a more efficient coupling method using a tapered optical fiber, ultralow threshold (~3 µW) green UC lasing was achieved in Er^3+^-doped microspheres^19^. However, the preparation of fluoride glasses is very demanding (in an inert atmosphere), and such glasses suffer from inferior chemical and thermal stabilities than oxide glasses.

Nano-glass composites (GCs) can be obtained by controlled thermal treatment of composition-designed precursor glasses (PGs). They offer combined benefits of high optical gain of the crystals and the favorable mechanical properties of glasses^20^. For example, fluoride-modified silicate (fluorosilicate) glasses, due to their unique amorphous phase separation, have been explored in hosting a number of fluoride nanocrystals (NCs, such as LaF_3_, NaYF_4_, KY_3_F_10_, etc.) upon thermal treatment^21–24^. Because of the preferential distribution of REs in the NCs, the photoluminescence quantum yield (PLQY) of UC emissions is significantly enhanced by up to three orders of magnitude. Therefore, GC-based microspheres could meet the requirement of high gain, robustness and long-term stability of microlasers, making them a new material solution for the development of the next generation of low-threshold UC microlasers^21^.

Although the study and development of REs-activated GC microspheres is still in its infancy, a few impressive achievements have already been made^25–27^. Compared with typical glass microspheres, GC microspheres containing Er^3+^-doped NaYF_4_ NCs were demonstrated to exhibit the lasing at 1557 nm with a much reduced (by ~60%) threshold (350 μW), and an increased (by 7 times) slope efficiency. The small difference in refractive indices (<0.06) between the host glass and the embedded NaYF_4_ NCs leads to low scattering loss, and guaranteeing a large value of *Q* > 10^5^^25^. In 2015, UC emissions were first realized in RE-activated GC microspheres incorporating LaF_3_ NCs^27^. However, there has been no report on visible UC lasing in GC microspheres, according to our knowledge^28^. There is continuous effort in exploring RE-doped GC materials with a reasonably large volume fraction of NCs, and a low scattering loss.

In this work, we demonstrated, for the first time, infrared-to-red, green and blue (RGB) visible UC lasing from GC microspheres incorporating Yb^3+^/Er^3+^ or Yb^3+^/Tm^3+^ doped KY_3_F_10_ and KMnF_3_ fluoride NCs. The microspheres were fabricated by melting of glass powers and the growth of the NCs was performed by thermal treatment, which exhibited high *Q* factors and strong UC emission. Comparisons are made for microspheres with and without REs-doped fluoride NCs with respect to the spectra profile, output power, lasing threshold and slope efficiency. The results show that GC microspheres are characterized by a reduced lasing threshold and an increased slope efficiency. Finally, the long-term stability of the proposed microlasers is verified, and potential applications as biosensors and highly sensitive refractometer are discussed.

## Results

### Fabrication of REs-doped nano-glass composite microspheres

**Fig. 1:**
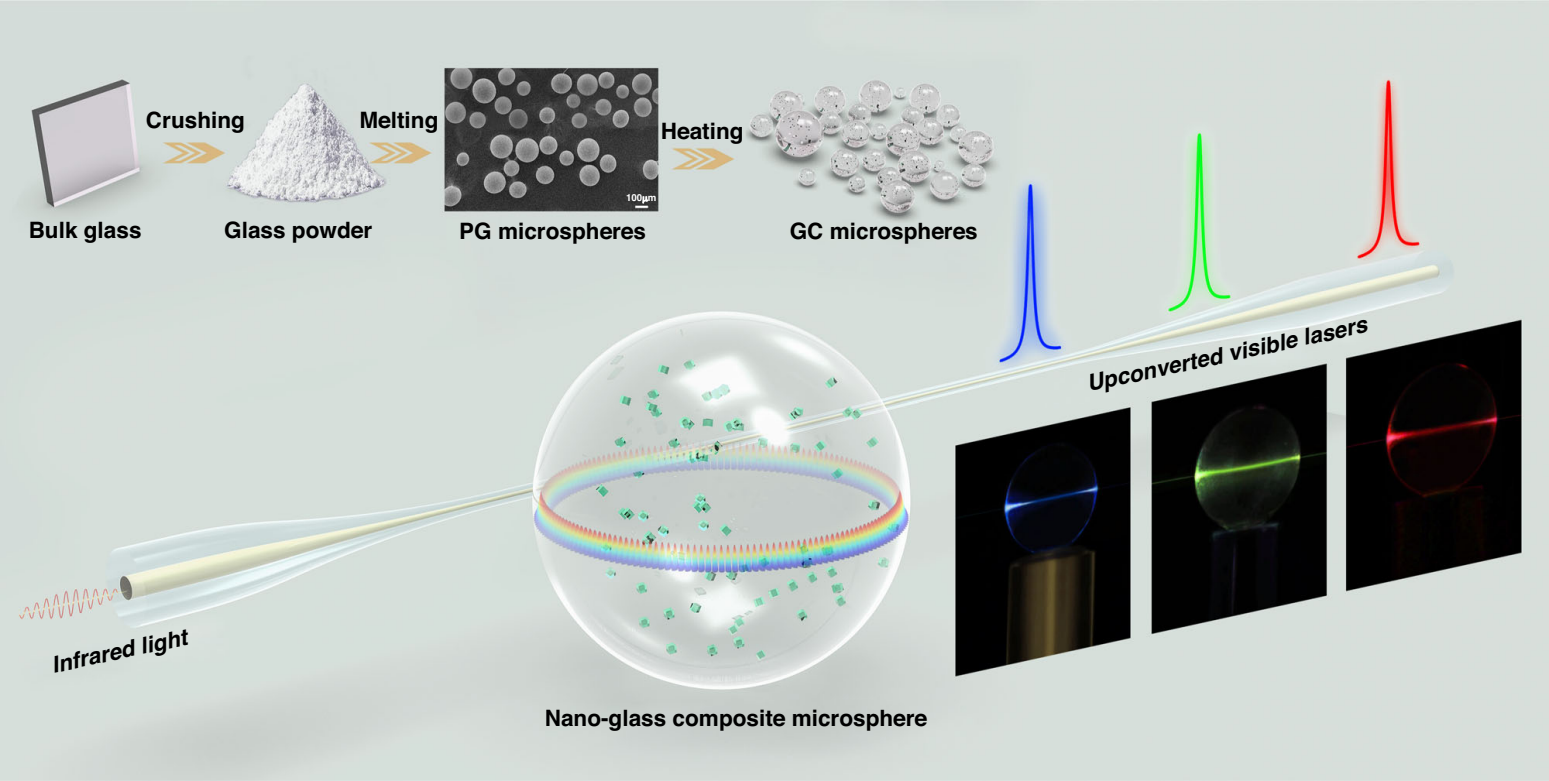
Nano-glass composite (GC) microsphere designed for infrared-to-upconverted visible lasers. Also shown is the batch production of GC microspheres by a powder melting and heating method

High *Q* factor (≥10^5^) GC microspheres with a spherical shape and a diameter *D* ranging from 0.9 to 200 μm were batch produced by a powder melting and heating method (Fig. 1, and Fig. S1, supporting information)^29^. The PG powers fabricated by grinding were melted in a vertical furnace to form glass microspheres, which were then heat-treated for crystallization. A size selection process was employed by using sieves with controlled pore sizes (Fig. S2, supporting information). Simulation shows that GC microspheres can be obtained with limited scattering losses if the particle size of the embedded NCs is below 50 nm, and the difference in refractive index between the NCs and the host glass is below 0.05 (Figs. S3 and S4, supporting information)^21^. The growth of KY_3_F_10_ and KMnF_3_ NCs in the microspheres was confirmed by XRD patterns (Fig. S5, supporting information), high-angle-annular-dark-field transmission electron microscopy (HAADF-STEM) and corresponding elemental mapping (Fig. S6, supporting information). The sizes of the KY_3_F_10_ and KMnF_3_ NCs are in the ranges of 20–50 nm and 8–20 nm, respectively (Fig. S7, supporting information)^23,24^.

### Experimental setup and simulation of coupling efficiency

**Fig. 2:**
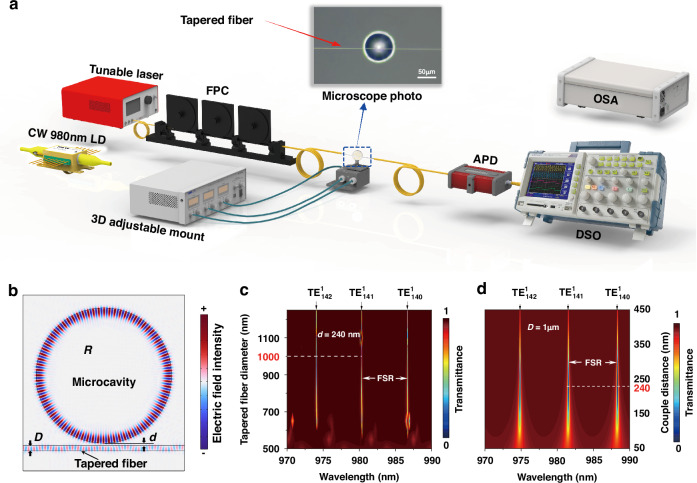
Experimental setup and simulation of coupling efficiency. **a** Schematic of CW laser pumped tapered fiber coupling system for the measurement of *Q* factor and laser output based on the microspheres. **b** Simulated distribution of the electric field of 980 nm light in the *x*-*y* plane of a microsphere of radius *R* coupled at a distance *d* to a tapered fiber of diameter *D*. **c** Dependence of transmission (indicated by the color bar) on the wavelength (*x*-axis) and the diameter of the tapered fiber at a fixed coupling distance *d* = 240 nm. **d** Dependence of transmission on the wavelength (*x*-axis) and the coupling distance *d* (*y*-axis) with a fixed tapered fiber diameter *D* = 1 μm. Transmission values (from 0 to 1) are indicated by the color bars

As shown in Fig. 2a, a tapered standard single mode fiber (SMF-28, Corning) was used for coupling with a microsphere. A tunable laser (Tunics-T100s-HP, Yenista) operating in the wavelength range of 1530–1580 nm with a narrow linewidth (300 kHz) was used to measure loaded *Q* factors of the microsphere. The light modulated by a fiber polarization controller (FPC) was evanescently coupled into the microsphere via the tapered fiber. The output power was also collected by the tapered fiber and sent to an avalanche photodetector (APD, APD310, Thorlabs) and a digital oscilloscope (DSO, MDO3054, Tektronix). The positions of the microsphere and the tapered fiber were adjusted by a three-axis piezo flexure stage (MDT630B, Thorlabs). The transmission spectra were monitored real time until critical coupling was achieved. For lasing measurement, the tunable laser, APD (and DSO) were replaced by a CW 980 nm LD and an optical spectrum analyzer (OSA, AQ6374E, Yokogawa), respectively.

The coupling efficiency between the tapered fiber and microsphere is simulated by the finite element method (Note 1, supporting information). As shown in Fig. 2b, a microsphere with a radius of *R* = 15 µm was side-coupled with a tapered fiber of diameter *D* at a coupling distance *d*. Figure 2c presents the dependence of transmission in the wavelength range of 970–990 nm on the diameter *D* of the tapered fiber at a fixed coupling distance *d* = 240 nm. When *D* = 1 µm, critical coupling was achieved at the resonant wavelengths of *λ* = 974.06 nm, 980.32 nm, and 986.66 nm, with a free spectral range (FSR) ≈ 6.3 nm. The WGMs thus observed are fundamental transverse electric field (TE) modes of ${TE}_{142}^{1}$, ${TE}_{141}^{1}$ and ${TE}_{140}^{1}$, where the superscript and subscript stand for the radial mode and angular mode numbers, respectively. By fixing the tapered fiber diameter *D* = 1 µm, the coupling distance *d* was found as *d* = 240 nm, Fig. 2d). The simulation also verifies that the tapered fiber plays a crucial role in exciting only the fundamental WGM modes, whereas the free space coupling could also excite higher order modes (Fig. S8, supporting information).

### WGM lasing from RE-doped GC microspheres

**Fig. 3:**
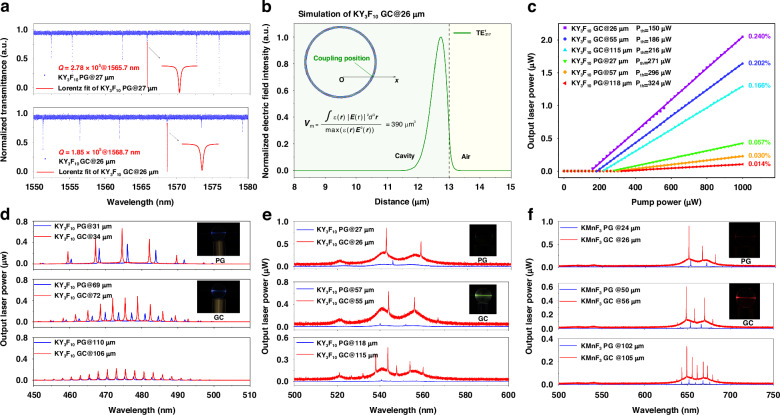
Infrared-to-visible lasing in nano-glass composite microspheres. **a** Loaded *Q* factors of a Yb^3+^/Er^3+^-doped PG (with a diameter of 27 μm, labeled by PG@27 μm) and a Yb^3+^/Er^3+^-doped KY_3_F_10_ GC@26 μm microspheres. Inset: selected WGMs fitted by a Lorentzian function. **b** Simulation of electric field intensity distribution of a selected laser mode in the GC microsphere. **c** Laser output power as a function of input pump power. **d**–**f** The size dependence of laser emission spectra of microspheres containing (**d**) Yb^3+^/Tm^3+^-doped KY_3_F_10_, (**e**) Yb^3+^/Er^3+^-doped KY_3_F_10_, and (**f**) Yb^3+^/Er^3+^-doped KMnF_3_ NCs at a pump power of 500 μW. Inset: Photos of visible lasing in the PG and GC microspheres

Upon excitation by a CW 980 nm LD at low powers, inhomogeneously broadened blue, green, and red UC emissions are observed (Fig. S9, supporting information), due to the ^1^G_4_ → ^3^H_6_ transition of Tm^3+^, ^2^H_11/2_/^4^S_3/2_ → ^4^I_15/2_ and ^4^F_9/2_ → ^4^I_15/2_ transitions of Er^3+^, respectively. The infrared-to-visible UC emissions occur by either a three-photon (for the blue) or a two-photon (for the green and red) absorption process (Fig. S10, supporting information). Compared with PG samples, the NCs embedded GC microspheres exhibit higher (by over an order of magnitude) PLQY, and longer decay lifetimes (*τ*) (Fig. S11, supporting information). These features are in favor of population inversion. The *Q* factor of the GC microspheres (for example, the Yb^3+^/Er^3+^-doped KY_3_F_10_ GC microsphere) is less than that of the corresponding PG samples (1.85 × 10^5^ vs. 2.78 × 10^5^). Although the GC microspheres exhibit a reduced *Q* due to the increased scattering loss (Fig. 3(a), and Fig. S12, supporting information), it is close to that of an optimized Er^3+^-doped NaYF_4_ GC microsphere (2–10 × 10^5^) reported previously^25^.

At pump powers above the threshold, several narrow lasing bands emerge (Fig. 3d–f). Compared with the PG samples, significantly increased laser output powers are observed for the GC microspheres under the same pump power. The laser output power of the individual laser mode decreases as the pump power is shared by several WMG modes. The number of laser mode increases with microsphere radius *R* because the wavelength spacing (*λ*_*m*_ - *λ*_*m*-1_) of neighboring WGMs (defined as FSR) shrinks with *R* according to (for a large *m* approximation): FSR ~ ${\lambda }_{m}^{2}$/2π*nR*, where *n* is the refractive index of the microsphere (1.54)^13^. All the WGMs are TE polarized, with their resonance wavelengths being well fitted by an asymptotic equation derived by Lam et al. ^13^ (Note 2, supporting information). The electric field intensity distribution of the fundamental mode was simulated in the radial and azimuthal directions of the microsphere (Fig. 3b). The lasing occurs extremely close to the microsphere surface, and is circulating along the equatorial plane. It decays exponentially outward of the interface between the microsphere and its surrounding medium.

As an example, when the diameter of the Yb^3+^/Er^3+^-doped KY_3_F_10_ GC microsphere increases from 26 μm to 115 μm, the laser threshold increases, and the input to output power conversion efficiency (viz., slope efficiency) decreases (Fig. 3c). The effective mode volume *V*_m_ is defined as the ratio of total electromagnetic energy stored in a specific mode to the maximum electromagnetic energy density (Fig. 3b). As *V*_m_ increases, the circulating intensity within the microcavity (*I*_circ_ = *P*_in_ × (*λ*_*in*_/2π*n*) × (*Q*/*V*_m_), where *P*_in_ is the input pump power of ~ 300 μW) decreases from 45.87 to 28.19 (GWm^−2^)^30^. Such high intracavity intensities are sufficient to establish population inversion of RE ions, which is otherwise difficult to achieve for non-cavity samples due to inherent fast relaxation rates at short wavelengths^9,31^. However, because the laser threshold is inversely proportional to *I*_circ_, it increases with microsphere diameter. This factor also accounts for the decrease in the slope efficiency. Compared with the PG sample (take the smallest size for example), the threshold of the GC microsphere is reduced by 44.6%, and the slope efficiency is increased by over four times. The optical conversion efficiency is also two orders of magnitude higher than that of UC microlasers based on nonlinear optical frequency conversion^32^.

For microcavities, the threshold mainly depends on the Purcell factor (∝ *Q*/*V*_m_)^16^. It means that the threshold is not only related to the *Q* factor but also to the mode volume *V*_m_. The simulation shows that as the diameter of microsphere increases, there is a rapid increase in the *Q* factor as a result of the exponential reduction of radiation loss. The *Q* factor tends to increase towards a saturation value when the diameter is over 40 μm (Fig. S13, supporting information). It also shows that the Purcell factor *Q*/*V*_m_ reaches the maximum value at a certain size ( ~ 20 μm). This result clearly confirms our experimental findings that the threshold of microlasers increases with the radius. Based on the simulated coupling coefficient *κ*^2^, and the experimentally determined *σ*_em_×*τ* = 8.8 × 10^-24 ^cm^2^ s^-1^ (Note 3, supporting information), the laser threshold *P*_th_ of the microsphere can be calculated according to *P*_th_ = 18*ln*(1/*L*_*oss*_)*hcλ*_p_/*σ*_em_×*τ*, where *σ*_em_ is the stimulated emission cross-section, *L*_*oss*_ is the total cavity loss per round trip, *c* is the speed of light, *h* is Planck’s constant, *λ*_p_ is the pump wavelength^33^. The threshold *P*_th_ was determined to be 83.3 μW. This value is smaller than the observed threshold of ~150 μW, which may be related to the coupling loss between the tapered fiber and the microsphere.

A comparison of laser performance is given for the reported UC microlasers (Table S1, supporting information). Apart from being the first study of RGB UC lasing from GC-based microspheres, there are several points worthy to be noted: (1) Our work is among the very few studies reporting slope efficiencies of UC microlasers. (2) For the blue lasing, the threshold of the Yb^3+^/Tm^3+^-doped KY_3_F_10_ GC microsphere is two orders of magnitude lower than that of the Tm^3+^-doped ZBALN microsphere (20 mW)^18^, and also lower than that of the silica microsphere coated by a Yb^3+^/Tm^3+^ doped polymethyl methacrylate film (~300 μW)^3^. (3) For the red lasing, the Yb^3+^/Er^3+^-doped KMnF_3_ GC microsphere is the first pure red-emitting microlaser due to the population regulation of Mn^2+^^23^. In contrast, the previously studied Yb^3+^/Er^3+^-doped microcavities exhibit multi-band visible emissions^3,34^. (4) The studied RGB UC microlasers exhibit low thresholds and higher slope efficiencies, and thus promising for color adaptation and full-color display, optical barcoding, and even white laser^35,36^.

## Stability of REs-doped GC microsphere and potential application

**Fig. 4:**
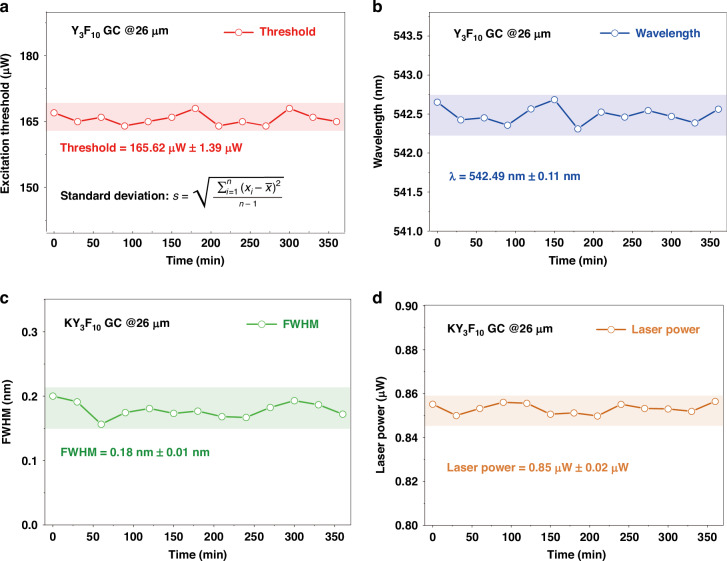
Stability test of the microlaser. Variations of (**a**) laser threshold, (**b**) wavelength, (**c**) FHWM and (**d**) output power of an Yb^3+^/Er^3+^-doped KY_3_F_10_ GC microsphere under continuous pumping by a CW 980 nm LD for a duration of 6 h

All the features of batch production, cost-effectiveness, and improved lasing performance of GC microspheres are attractive for applications. This application is further benefited from the long-term stability of GC microspheres (Fig. 4), which is an important issue for UC lasers since high pump intensities are often required for step-wise two- or multi-photon absorption^9^. Our all-solid-state microsphere laser system operate with high stability at RT for more than 6 h under continuous pumping. As shown in Fig. 4, the fluctuations in laser threshold, wavelength, FHWM and output power of the Yb^3+^/Er^3+^-doped KY_3_F_10_ GC microsphere are within, respectively, 0.84%, 0.02%, 5.6% and 2.4%. Moreover, there is only a slight change in the *Q* factor of a GC microsphere stored at ambient atmosphere in a clean room for one year, confirming again stable performance of WGM lasing in terms of spectra profile and output power are preserved (Fig. S14, supporting information). In contrast, CW lasing based on semiconductor NCs is not stable for an operation duration only 1 h^37^. For polymer-based microspheres, irreversible composition or shape change was found previously when exposed to continuous high-power pumping, which poses serious obstacles for long-term applications^6^.

From the application point of view, the RE-doped microlasers can provide a 10^4^-fold narrower resonance linewidth than a passive microcavity with respect to the transmission spectrum^38^. This, together with the enhanced signal-to-noise ratio of laser lines, could be desirable for enhancing the sensitivity of biosensors^17^. Previous biosensors were based on microlasers emitting in the NIR wavelengths (780–1550 nm), where water has a relatively strong absorption and therefore severely limits the sensitivity of sensors^7^. Since water absorption is greatly reduced in the visible, interests have been gravitated towards visible microlasers. Considering that both the pump and lasing wavelengths lie in the biological transparent and low water absorption window (450–980 nm)^14^, our UC microlasers could provide crucial light sources for bio/chemical sensors^39^. The multiple-wavelength microlasers (c.f. Fig. 3) have been used to reduce the detection errors induced by the position-dependent change in frequency splitting^40^. Moreover, the construction of arrays of microspheres with surface functionalized via silanization and bio-conjugation may open a gate to a miniatured detection chip^41^.

For single nanoparticle sensing, the sensitive detection of small changes in refractive index (RI) is highly demanded. Following a previous theoretical work^33^, we have evaluated the RI sensitivity of the studied microspheres. It was calculated that the GC microspheres could achieve a lasing linewidth ∆*ω* = 1.44 kHz. Based on the laser linewidth, the detection limit of the external refractive index *n*_ex_ was obtained according to ∆*n*_ex_/*n*_ex_ = ∆*ω*/*ω*. The GC microsphere could detect an index change of the order of ~10^-8^ in water solution. As compared with conventional integrated optical waveguide sensors, this result represents a several orders of magnitude decrease in detection limit.

## Discussion

High-*Q* (≥10^5^) GC microspheres incorporating Yb^3+^/Tm^3+^ (or Yb^3+^/Er^3+^) doped fluoride NCs have been fabricated by using the powder melting followed by heating induced crystallization. The limited particle size of the NCs and the small difference in the refractive index between the NCs and the glass matrix account for the retention of high *Q* factor. Leveraging the tapered fiber-microsphere coupling architecture and the strong confinement of pump light into the fundamental WGMs of the microsphere, low threshold red, green, blue UC lasing are obtained, for the first time, based on GC-based microspheres. The fluoride NCs grown in the glass microsphere provides a favorable low-phonon energy environment for RE ions and a larger figure-of-merit (*σ*_*em*_×*τ*), resulting in the significantly reduced (up to 45%) threshold and enhanced (more than four times) slope efficiency. The robustness and chemically inert nature of the GC microspheres secures their long-term stability such that the laser performance barely changes under the uninterrupted pumping for a duration of over 6 hours. The high *Q* factor and stable lasing are maintained even one year after the fabrication of the microsphere. The demonstration of RGB UC microlasers would also facilitate the development of white lasers (by simultaneous doping of Yb^3+^, Tm^3+^, and Er^3+^ in a proper ratio). Our work unveils a largely uncharted area of visible microlasers based on GC microspheres, and showcases a useful tool that are highly demanded in on-chip spectroscopy, optical barcoding, chemical and physical sensing, as well as cavity quantum electrodynamics seeking for miniaturized, robust, low threshold, and narrow band laser sources.

### Experimental section

#### Fabrication of the RE activated GC microspheres

Three fluorosilicate bulk glasses were prepared by the conventional melt-quenching method with the compositions (in mol%) of 50SiO_2_-25YF_3_-25KF-1.0YbF_3_-0.2TmF_3_ (for blue laser), 50SiO_2_-25YF_3_-25KF-1.0YbF_3_-0.2ErF_3_ (for green laser) and 70SiO_2_-15MnF_2_-15KF-1.0SnCl_2_-1.0YbF_3_-0.2ErF_3_ (for red laser). High purity (4 N) SiO_2_, YF_3_, KF, MnF_2_, SnCl_2_ and (3 N) YbF_3_, ErF_3_, TmF_3_ were used as the raw materials. The use of SnCl_2_ is to create a reducing atmosphere to avoid the oxidation of Mn^2+^ to Mn^3+^. In a typical batch, approximately 30 g of raw materials was mixed completely and melted in a covered platinum crucible at 1550 °C for 20 mins in air. The melt was cast onto a stainless-steel plate, and then annealed at 450 °C for 3 h. After naturally cooling to ambient temperature, bulk precursor glasses (PGs) were obtained.

A powder floating method was used to prepare microspheres (Fig. S1, supporting information). Powders of glass were floated in a vertical furnace set at 1500 °C for a duration of about 0.5 s. PG microspheres thus obtained were collected in a petri dish installed in a water bath at the bottom of the furnace. GC microspheres were then obtained by heating the corresponding PG microspheres at 750 °C for 5 h for the blue and green lasers, and at 550 °C for 10 h for the red laser. For laser experiment, the microsphere was attached on the tip of an optical fiber that was fabricated by cutting a piece of fiber taper (waist diameter of ∼20 μm) in the tapering region.

#### Characterizations

X-ray diffraction (XRD) patterns were recorded using an X-ray diffractometer (D/MAX 2550VB/PC, Rigaku Corproation) with Cu-Kα irradiation. Transmission electron microscopy (TEM) images and high-angle-annular-dark-field scanning TEM (HAADF-STEM) images were captured using a FEI Talos F200X microscope operating at an acceleration voltage of 200 kV. The TEM samples were processed by conventional mechanical polishing and ion beam milling techniques (PIPS II system from GATAN) to about 50 nm in thickness. Optical transmission spectra were measured by using a Perkin-Elmer Lambda 1050 + UV–NIR spectrophotometer. Photoluminescence (PL) and decay spectra were recorded using a FLS1000 Fluorescence spectrometer (Edinburgh Instruments). Morphology of microspheres was observed by scanning electron microscopy (SEM, Tescan Mira 3 XH, Tescan). The simulation of the local EM field in the GC microspheres was carried out by a finite-element method using COMSOL Multiphysics (COMSOL Inc.) software.

## Supplementary information

Supplementary Information
