# Correction: Robust low threshold full-color upconversion lasing in rare-earth activated nanocrystal-in-glass microcavity

**DOI:** 10.1038/s41377-025-01765-6

**Published:** 2025-02-25

**Authors:** Zhigang Gao, Lugui Cui, Yushi Chu, Luyue Niu, Lehan Wang, Rui Zhao, Yulong Yang, Xiaofeng Liu, Jing Ren, Guoping Dong

**Affiliations:** 1 College of Physics and Electronic Engineering, Taishan University, 271021 Taian, China; 2 Key Laboratory of In-fiber Integrated Optics of Ministry of Education, College of Physics and Optoelectronic Engineering, Harbin Engineering University, 150001 Harbin, China; 3 School of Materials Science and Engineering, Zhejiang University, Hangzhou, China; 4 State Key Laboratory of Luminescent Materials and Devices, and Guangdong Provincial Key Laboratory of Fiber Laser Materials and Applied Techniques, School of Materials Science and Engineering, South China University of Technology, 510640 Guangzhou, China

**Keywords:** Lasers, LEDs and light sources, Optical materials and structures

Correction to: *Light: Science & Applications*

10.1038/s41377-024-01671-3, published online 02 January 2025

After publication of the article, it was brought to our attention that figure 3 need to be amended.

The incorrect figure 3:
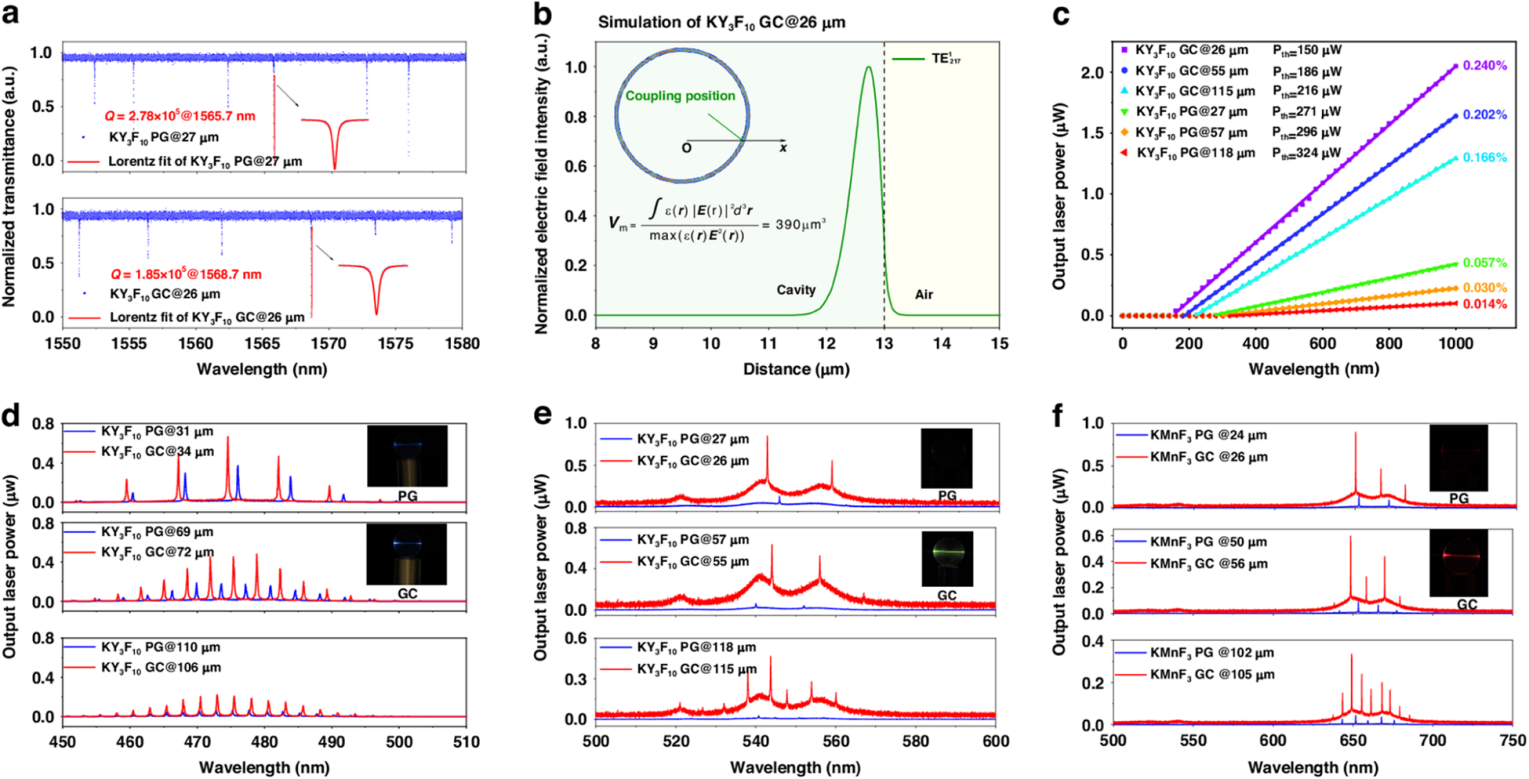


The correct figure 3:
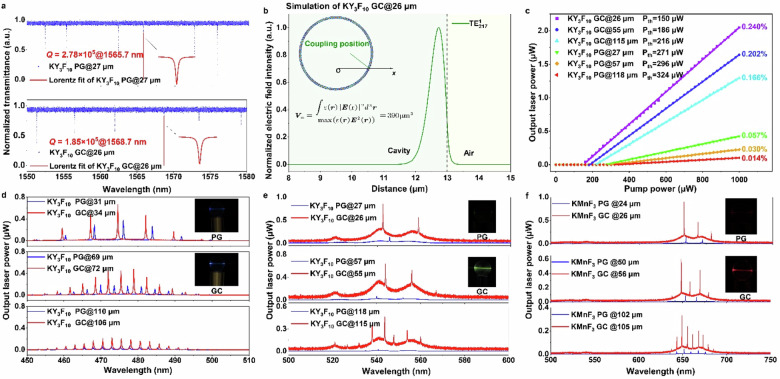


The original publication has been corrected.

