# Supplementary material for: Robust low threshold full-color upconversion lasing in rare-earth activated nanocrystal-in-glass microcavity
Source: Light Sci Appl. 2025 Jan 2;14:14. doi: 10.1038/s41377-024-01671-3 (PMC11693753; doi:10.1038/s41377-024-01671-3)
Supplement: Supplementary file 1 — Supplementary Information [file 41377_2024_1671_MOESM1_ESM.pdf]

Supporting Information for

Robust low threshold full-color upconversion lasing in rare-earth activated nanocrystal-in-glass microcavity

Zhigang Gao [1], Lugui Cui [2], Yushi Chu [2], Luyue Niu [2], Lehan Wang [2], Rui Zhao [1], Yulong Yang [2], Xiaofeng Liu [3], Jing Ren [2]✉ and Guoping Dong [4]✉

[1] *College of Physics and Electronic Engineering, Taishan University, Taian 271021, China*

[2] *Key Laboratory of In-fiber Integrated Optics of Ministry of Education, College of Physics and Optoelectronic Engineering, Harbin Engineering University, Harbin 150001, China*

[3] *School of Materials Science and Engineering, Zhejiang University, Hangzhou, China*

[4] *State Key Laboratory of Luminescent Materials and Devices, and Guangdong Provincial Key Laboratory of Fiber Laser Materials and Applied Techniques, South China University of Technology, Guangzhou 510640, China*

Correspondence: Jing Ren (*ren.jing@hrbeu.edu.cn*); Guoping Dong (*dgp@scut.edu.cn*)

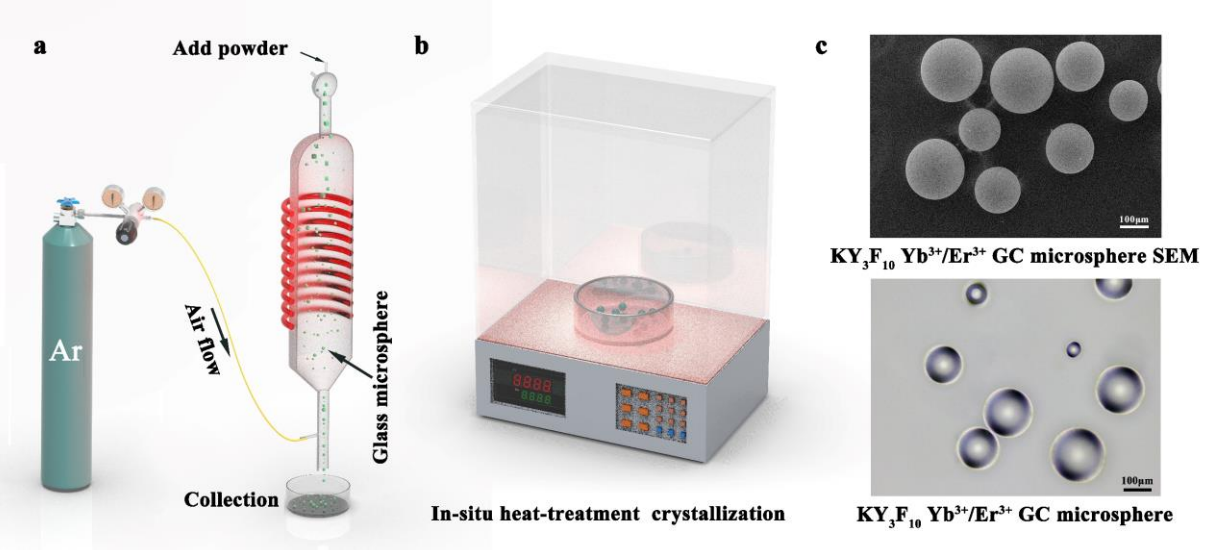

Fig. S1 a Schematic of the powder floating method used to prepare precursor glass (PG) microspheres. The bulk glass was first crushed into powders. The raw materials powders were added from the top of the furnace, melting occurs when they fall through the heating zone. As a result of the surface tension, the formation of the microspheres of the PG occurred. Finally, size selection for microspheres were carried out by using a sieve with a specific size. **b** Nano-glass composite (GC) microspheres were obtained by heat-treatment induced crystallization of the PG microspheres; **c** Scanning electron microscope (SEM) images of the as-made GC microspheres.

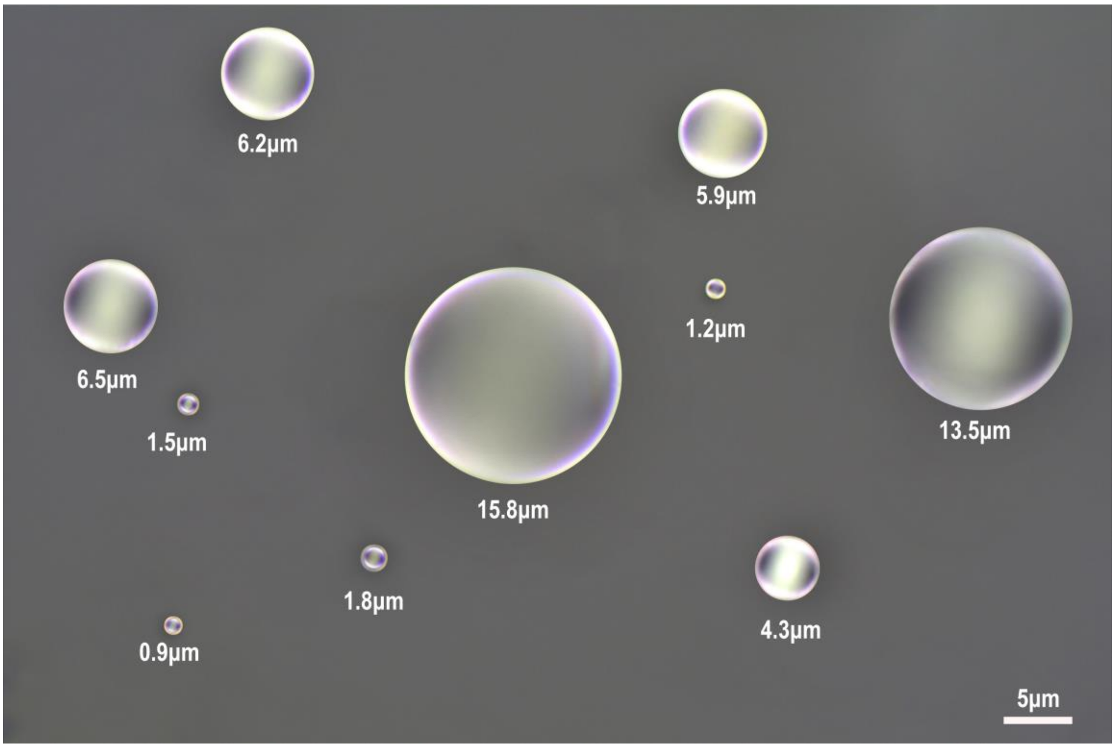

Fig. S2 GC microspheres with different sizes. To select the size of the microspheres, sieves with different pore sizes (e.g., 100 mesh, 300 mesh, 500 mesh, etc.) were used.

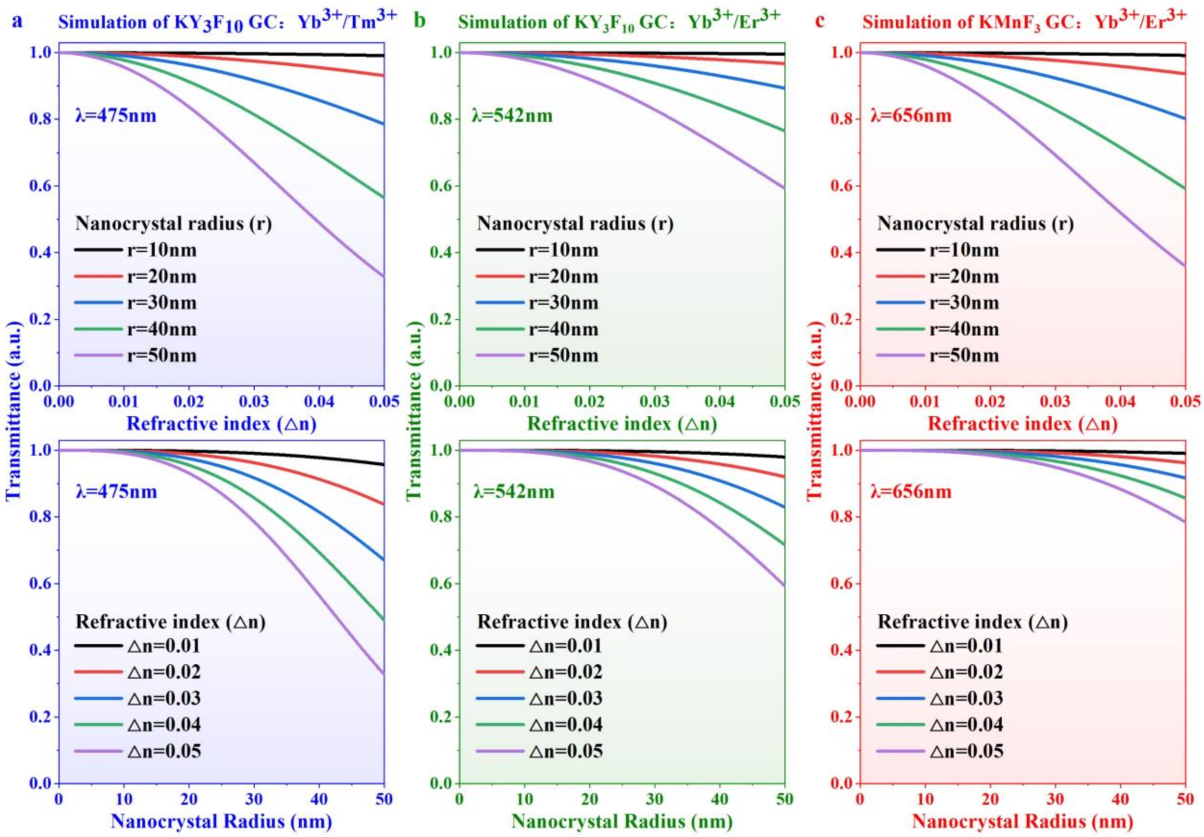

Fig. S3 Theoretical transmittance of the GCs incorporating **a** Yb^{3+}/Tm^{3+}, **b** Yb^{3+}/Er^{3+} doped KY_3F_{10} NCs, and **c** Yb^{3+}/Er^{3+} doped $KMnF_3$ NCs. The first row shows the dependence of transmittance on the refractive index difference between the NCs and the glass matrix for different particle radii of NCs, and the second row shows dependence of transmittance on the particle size of NCs at different refractive index differences.

The light scattering by the nano-glass composites can be described by using the Rayleigh scattering model, and the theoretical transmittance can be obtained as [1]:

$$T = e^{-\varepsilon L} = e^{\frac{-32 m L \pi^4 n^4 r^3}{\rho \lambda_0^4}\left(\frac{n^2 - n_0^2}{n^2 + 2n_0^2}\right)} \qquad \text{(S1)}$$

where, ε is the scattering loss, L is the optical path, m is the total mass of the embedded NCs, ρ is the packing density of NCs in the glass matrix, r is the radius of NCs, n_0 and n are the refractive indices of the glass matrix and the NCs respectively, λ_0 is the wavelength of light in vacuum. The numerical model of Rayleigh scattering was constructed by function analysis using COSMOL Multiphysics software. The following parameters were used in the calculation: The refractive indices of the glass matrix, KY_3F_{10} and $KMnF_3$ NCs are 1.52, 1.48, 1.45, respectively [2]. The volume fraction ρ of KY_3F_{10} and $KMnF_3$ NCs is 20%, and their respective densities are 3.10 g/cm^3, and 2.69 g/cm^3.

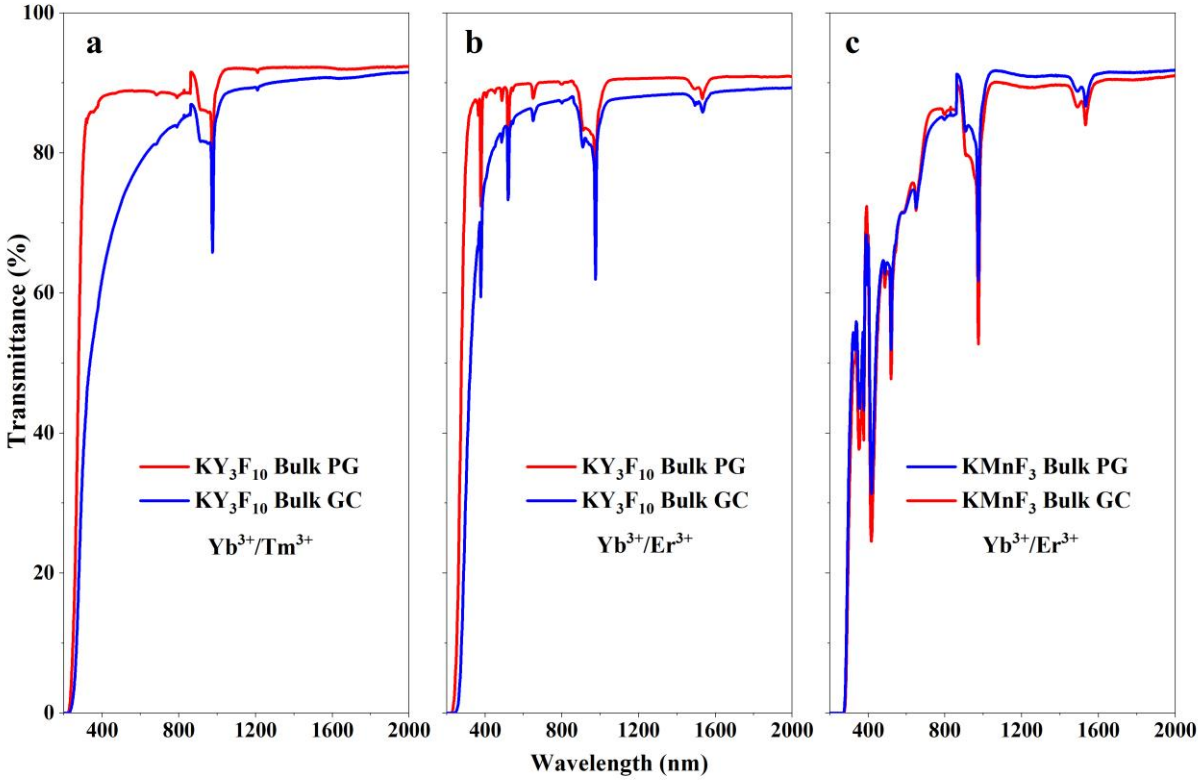

Fig. S4 Transmission spectra of samples. **a-b** PG and the corresponding GC samples incorporating **a** Yb^{3+}/Tm^{3+}, **b** Yb^{3+}/Er^{3+}doped KY_3F_{10}, and **c**Yb^{3+}/Er^{3+} doped $KMnF_3$ NCs. The transmittance decreases in the short wavelength region because of the Rayleigh scattering induced by the NCs. At the output wavelength of the lasers, the transmittance exceeds 80%. The main reason for the high transmittance after crystallization are the small size of the crystals, small refractive index mismatch between the crystals and glass matrix, and the absence of birefringence because of the isotropic nature of the crystals.

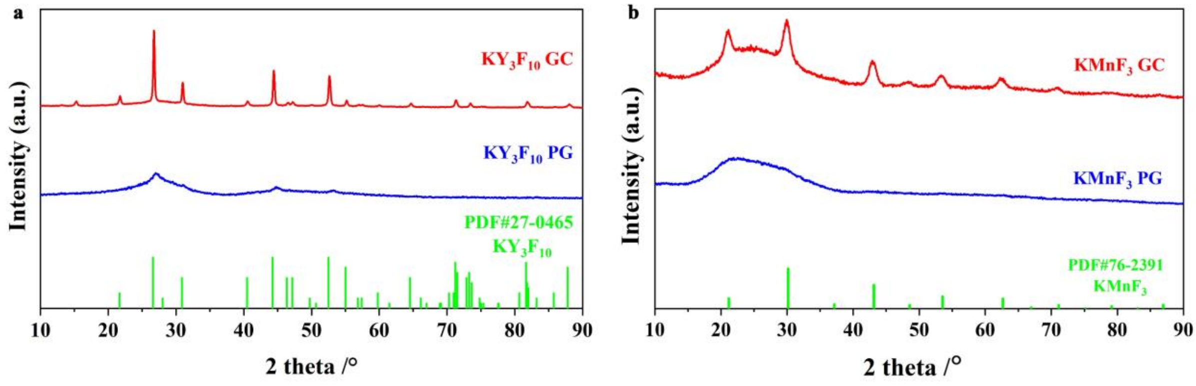

Fig. S5 X-ray diffraction (XRD) patterns of the PG and the corresponding GC samples incorporating **a** KY_3F_{10} and **b** $KMnF_3$ nanocrystals. The PG samples display a typical amorphous hump, while several sharp diffraction peaks attributing to the KY_3F_{10} (JCPDS No. 27-0465) and $KMnF_3$ crystals (JCPDS No. 76-2391) appear in the GC samples.

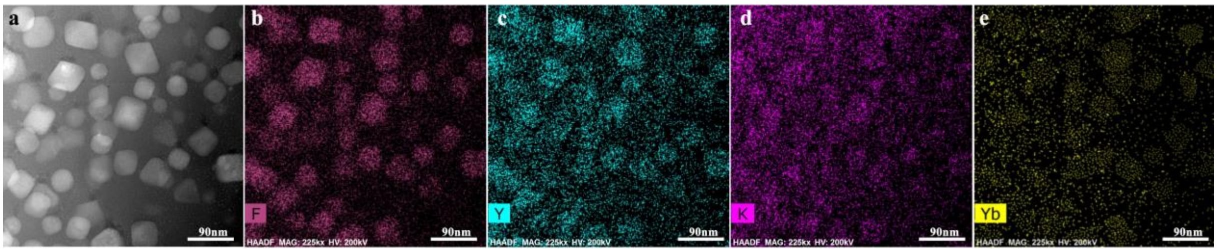

Fig. S6 a HAADF-STEM images of the microspheres, and **b-e** elemental mappings with the concentration reflected by the brightness in color.

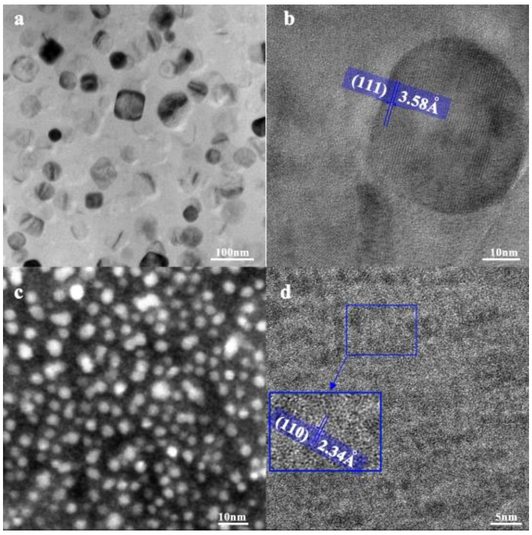

Fig. S7 a, c Transmission electron microscope (TEM) images of the GCs incorporating **a** KY_3F_{10} and **c** $KMnF_3$ crystals. The sizes of KY_3F_{10} and $KMnF_3$ NCs are among 20~50 nm and 8~20 nm respectively, and these NCs are uniformly dispersed in the amorphous glass matrix. **b, d** High-resolution transmission electron microscope (HR-TEM) images of a single **b** KY_3F_{10} and **d** $KMnF_3$ nanocrystal. The crystal lattices with a d-spacings of 3.58 and 2.34 Å match well with the (111) planes of the KY_3F_{10} and $KMnF_3$ crystals, respectively.

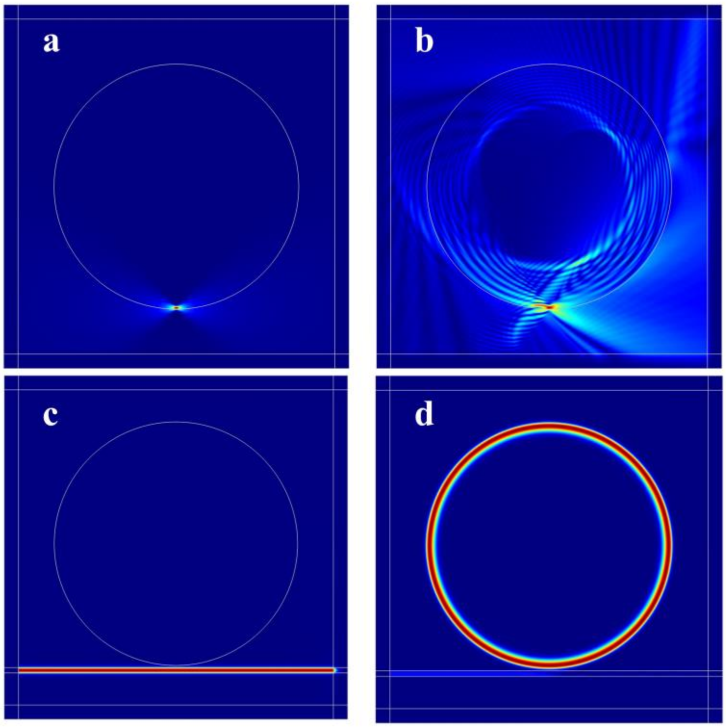

Fig. S8 (a) Field distribution when a background Gaussian beam is transmitted from left to right and focused on the edge of a microsphere in free space. (b) Field distribution of scattered light for a microsphere under Gaussian beam excitation. Field distribution under (c) non-resonant and (d) on-resonance conditions when a tapered fiber is coupled to a microsphere.

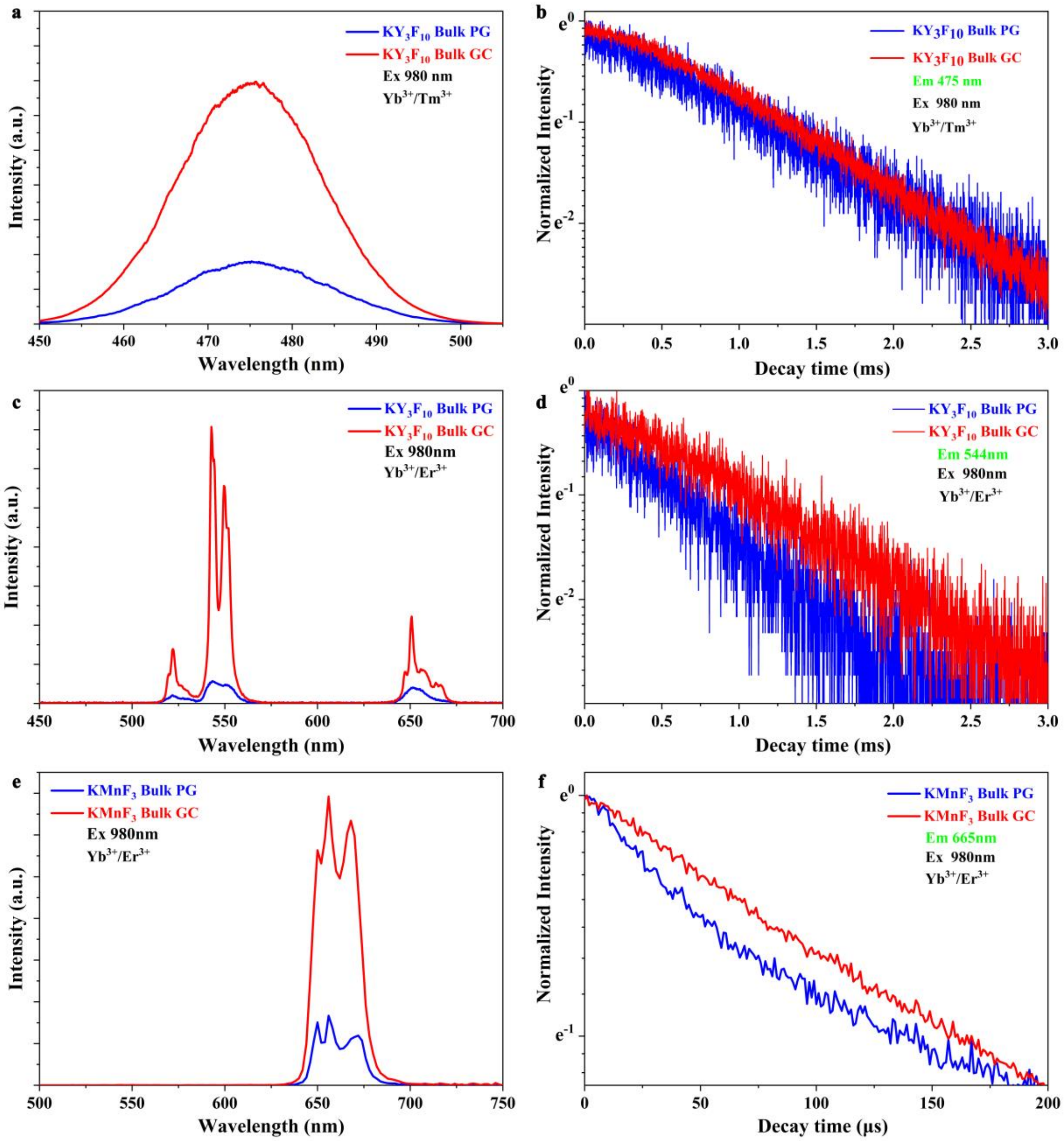

Fig. S9 Upconversion luminescence spectra of the PG and GC samples under excitation by a 980 nm laser diode. **a** PG and the corresponding GC incorporating Yb^{3+}/Tm^{3+} doped KY_3F_{10} NCs, **c** PG and the corresponding GC incorporating Yb^{3+}/Er^{3+} doped KY_3F_{10} NCs, **e** PG and the corresponding GC incorporating Yb^{3+}/Er^{3+} doped $KMnF_3$ NCs. In all cases, as compared to the PG samples, the UCL intensities of the GC samples are much stronger. The corresponding decay curves of the upconversion emissions of samples are shown in **b**, **d** and **f**. The decay rates of GCs are slower than those of PGs due to the change of the local environment for rare earth ions as a result of the transition from amorphous glassy phase of high-lattice phonon energies to the crystalline phase with much lower phonon energies.

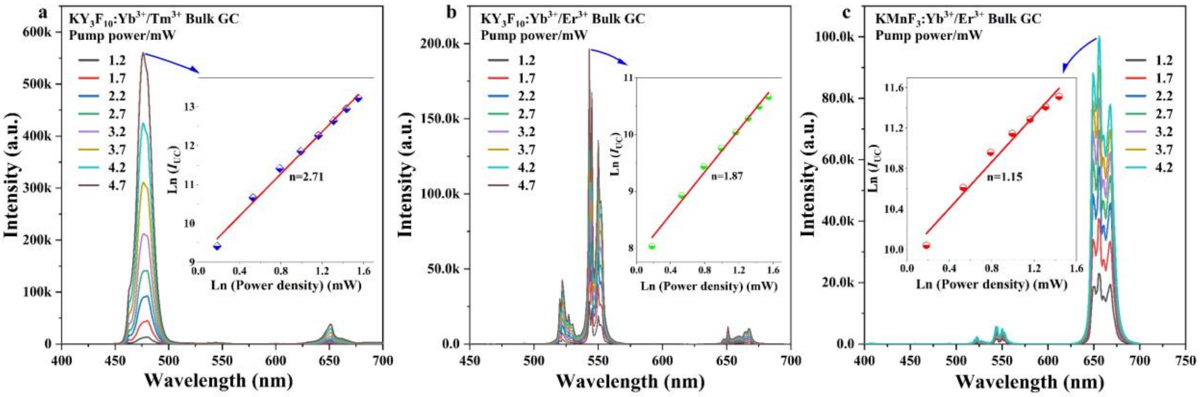

Fig. S10 Upconversion luminescence spectra of **the** 475 nm blue emission from the PG and the corresponding GC incorporating Yb^{3+}/Tm^{3+} doped KY_3F_{10} NCs, **b** the 544 nm green emission from the PG and the corresponding GC incorporating Yb^{3+}/Er^{3+} doped KY_3F_{10} NCs, and **c** the 665 nm red emission from the PG and the corresponding GC incorporating Yb^{3+}/Er^{3+} doped $KMnF_3$ NCs under different excitation powers (P_{pump}). Insets: Plots of Log(P_{pump}) versus Log(I_{UC}), and fits to a linear function. I_{UC} stands for the peak intensity of the upconversion luminescence. The results indicate that the blue and red UC emissions occur through a two-photon absorption process, while the red emission is dominated by a three-photon absorption process.

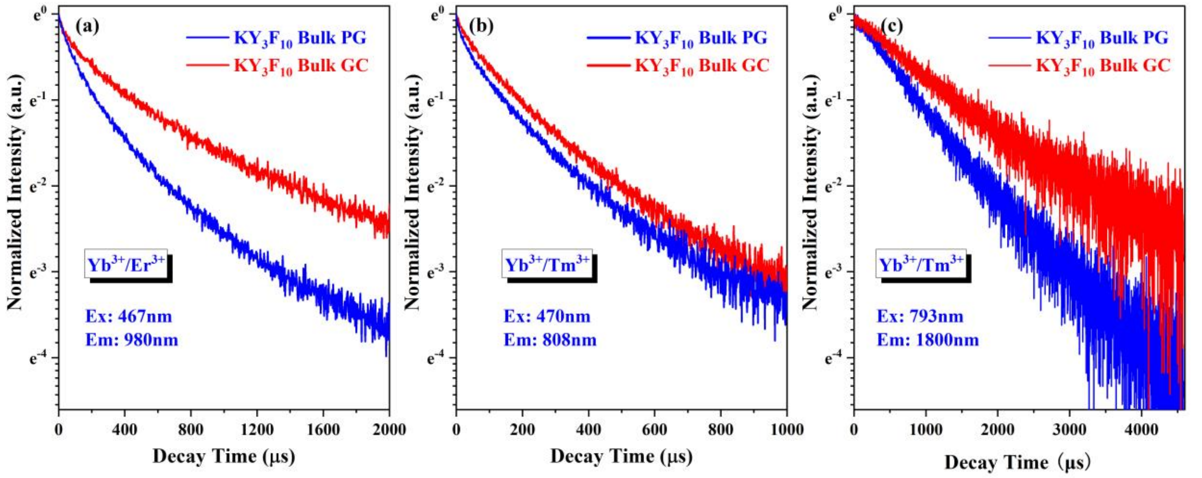

Fig. S11 PL decay curves of samples. **a** Decay curves of the 980 nm emission from the PG and the corresponding GC incorporating Yb^{3+}/Er^{3+} codoped KY_3F_{10} NCs. Decay curves of the **b** 808 nm and **c** 1800 nm emissions from the PG and the corresponding GC incorporating Yb^{3+}/Tm^{3+} codoped KY_3F_{10} NCs. In all cases, the PL decay times of GCs are longer than those of PGs, benefiting the promotion of upconversion luminescence.

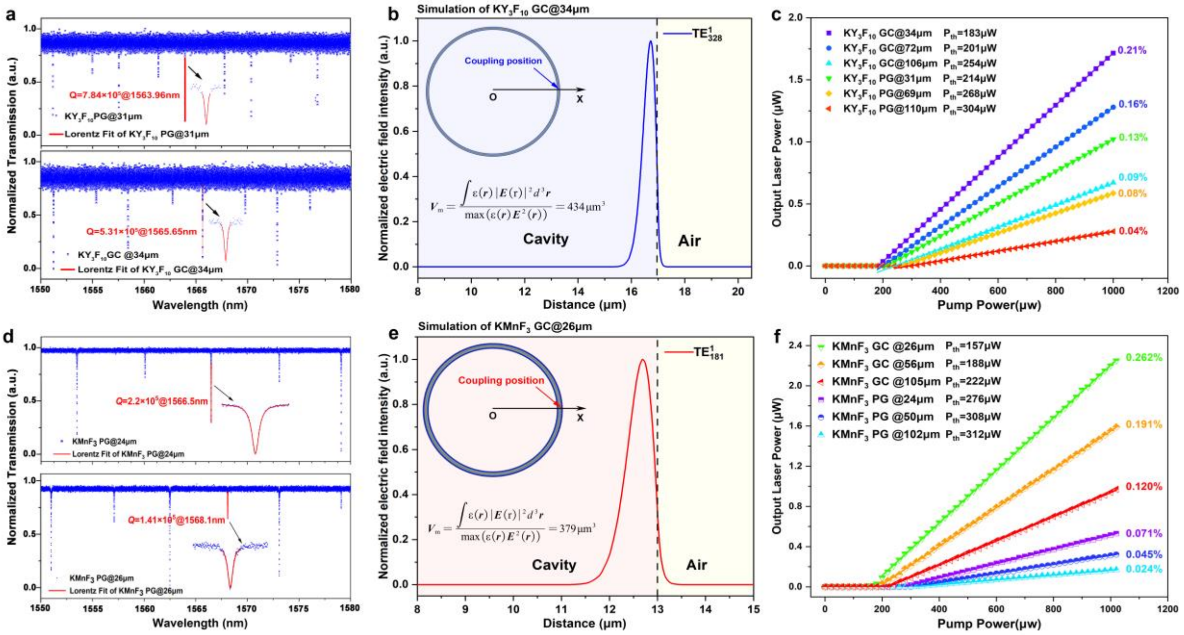

$$V_m = \frac{\int \varepsilon(r)\,|E(r)|^2 d^3r}{\max(\varepsilon(r)E^2(r))} = 434\,\mu m^3$$

$$V_m = \frac{\int \varepsilon(r)\,|E(r)|^2 d^3r}{\max(\varepsilon(r)E^2(r))} = 379\,\mu m^3$$

Fig. S12 **a** Loaded *Q* factors of a Yb^{3+}/Tm^{3+}-doped PG (with a diameter of 31 μm, labeled by PG@31 μm) and a Yb^{3+}/Tm^{3+}-doped KY_3F_{10} GC@34 μm microspheres. Inset: selected WGMs fitted by a Lorentzian function; **b** Simulation of electric field intensity distribution of a selected laser mode in the Yb^{3+}/Tm^{3+}-doped KY_3F_{10} GC@34 μm microsphere; **c** Laser output power as a function of input pump power; **d** Loaded *Q* factors of a Yb^{3+}/Er^{3+}-doped PG (with a diameter of 24 μm, labeled by PG@24 μm) and a Yb^{3+}/Er^{3+}-doped $KMnF_3$ GC@26 μm microspheres. Inset: selected WGMs fitted by a Lorentzian function; **e** Simulation of electric field intensity distribution of a selected laser mode in the Yb^{3+}/ Er^{3+}-doped $KMnF_3$ GC@26 μm microsphere; **f** Laser output power as a function of input pump power.

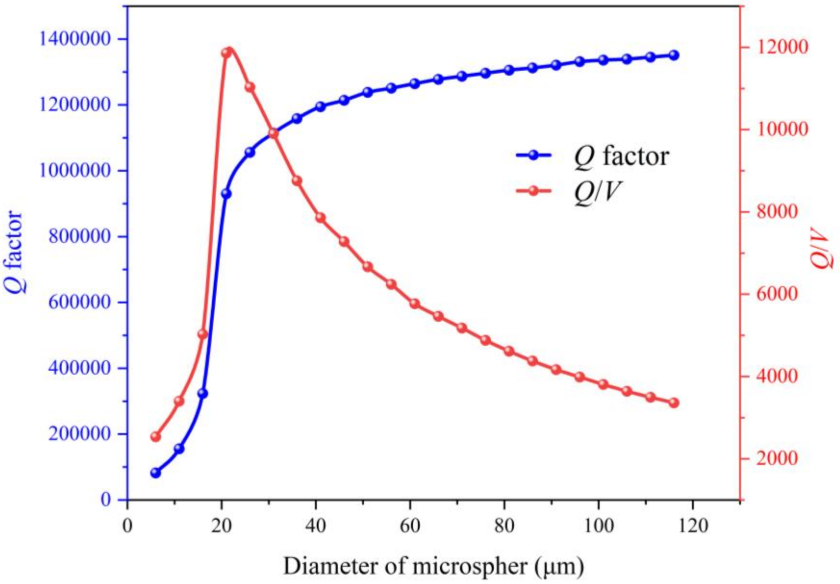

Fig. S13 Dependence of quality factor Q and Purcell factor (∝*Q*/V) on the diameter of a microsphere.

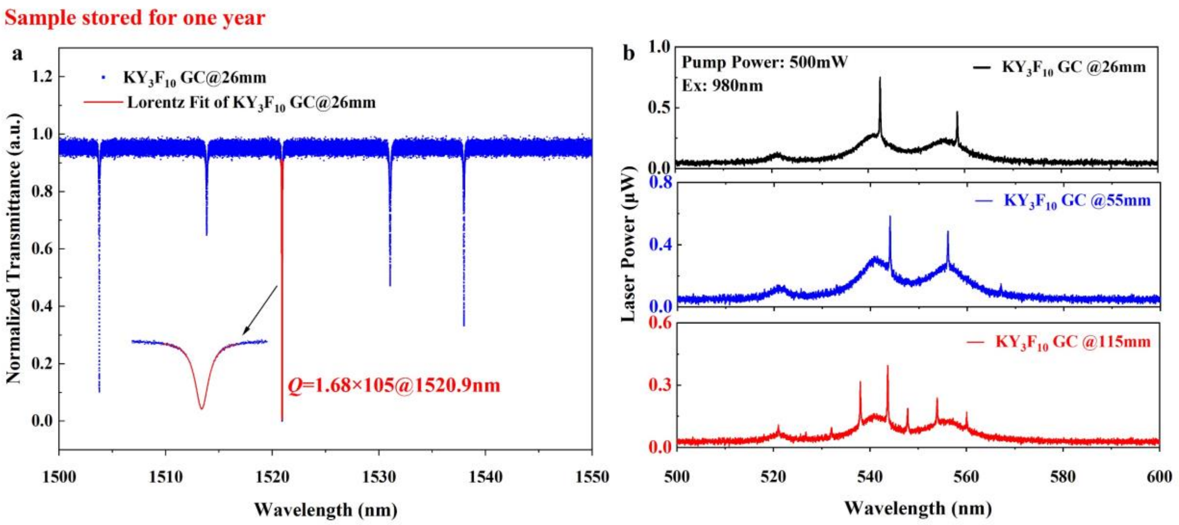

Fig. S14 a Quality factor, and **b** laser spectra measured of a Yb^{3+}/Er^{3+}-doped GC microsphere stored at ambient atmosphere for one year.

Note1: Simulation method

Simulation is performed by using the commercial finite element software COSMOL Multiphysics for model construction, calculation and result processing. In the simulation, the characteristic frequency of the wave optics module was used to calculate the quality factor (Q), resonance frequency, and corresponding electric field distribution of WGM microspheres. Transmittance spectrum and coupling efficiency of WGM microspheres were calculated using frequency domain methods. It should be noted that due to the large size of the microsphere, a symmetric structure was used in the analysis of its characteristic frequencies and frequency domain to reduce the degrees of freedom in model analysis. Perfect electrical conductor boundary conditions were applied on the symmetry plane to ensure that the model is in standard TE mode.

Note 2: Calculation of resonant wavelengths of WGM

All the WGMs shown in Figure 3(c) can be ascribed to transverse electric (TE) modes, with the resonance wavelengths well fitted by the asymptotic equation: [3-4]

$$\lambda^{-1}(R,n_1,n_r,q,m)=\frac{1}{2\pi Rn_1}\left[m+\frac{1}{2}+2^{-\frac{1}{3}}\alpha(q)\left(m+\frac{1}{2}\right)^{\frac{1}{3}}-\frac{L}{(n_r^2-1)^{\frac{1}{2}}}+\frac{3}{10}2^{\frac{2}{3}}\alpha^2(q)\left(m+\frac{1}{2}\right)^{-\frac{1}{3}}-2^{-\frac{1}{3}}\left(n_r^2-\frac{2}{3}L^2\right)\frac{\alpha(q)\left(m+\frac{1}{2}\right)^{-\frac{2}{3}}}{(n_r^2-1)^{\frac{3}{2}}}\right] \quad \text{S(2)}$$

where λ is the resonant wavelength, R is the microsphere radius, n_1 is the refractive index of the microcavity, $n_r = n_1/n_2$ with n_2 the refractive index of the surrounding medium, $L=n_r$ for TE modes, $\alpha(q)$ are the roots of the Airy function, and q as the radial mode number, respectively. There are slightly differences between the theoretical and experimental wavelengths, which is ascribed to the ellipsoid of the microsphere, the relative position of the microsphere and the coupling tapered fiber and the ambient temperature fluctuation.

Note 3: Judd-Ofelt (JO) analysis and calculation of $\sigma_{em}\times\tau$

To understand the lower thresholds of GC microspheres, a figure of merit, i.e., the product of stimulated emission cross-section σ_{em} with radiative decay time τ, was calculated by using the structure-sensitive theoretical analysis based on Judd-Ofelt (JO) intensity parameters (Ω_2, Ω_4,

Ω_6). The JO intensity parameters (Ω_2, Ω_4, Ω_6) were obtained by fitting the experimentally determined oscillator strength f_{exp} against the theoretical ones [5-6]. Based on the JO parameters, the spontaneous transition probability A_{rad} can be expressed as:

$$A_{rad} = A_{ed} + A_{md} = [\frac{1}{4\pi\varepsilon_0}]\frac{64\pi^4 e^2}{3h(2J+1)\bar{\lambda}^3}[\frac{n(n^2+2)^2}{9}S_{ed} + n^3 S_{md}]$$
$$= 7.235\times10^{31}\times\frac{1}{(2J+1)\bar{\lambda}^3}\times[\frac{n(n^2+2)^2}{9}S_{ed} + n^3 S_{md}] \quad S(3)$$

where A_{ed} (s^{-1}) and A_{md} (s^{-1}) are the electric-dipole and magnet-dipole transition probabilities, respectively, and S_{ed} (cm^2) and S_{md} (cm^2) are the electric-dipole and magnet-dipole line strengths according to:

$$S_{ed} = \sum_{t=2,4,6}\Omega_t\left|\left\langle aJ\left\|U^{(t)}\right\|bJ'\right\rangle\right|^2 \quad S(4)$$

$$S_{md} = \frac{1}{4m^2c^2}\left|\left\langle aJ\left\|L+2S\right\|bJ'\right\rangle\right|^2 \quad S(5)$$

The emission cross-section, σ_{em}, can be calculated from the measured emission spectra according to the Fuchtbauer-Ladenburg equation:

$$\sigma_e = \frac{\lambda^4}{8\pi cn^2}\frac{A_{rad}}{\Delta\lambda_{eff}} \quad S(6)$$

where $\Delta\lambda_{eff}$ is the effective fluorescence bandwidth that can be calculated by dividing the area of the emission band by its peak height.

Table S1 Comparison of laser performance of infrared-to-visible UC WGM lasers

Year	Cavity design	Pump parameter	Wavelength (nm)	FWHM (nm)	Threshold	Q value	Slope efficiency
**1999** [7]	Tm^{3+}-doped ZBLAN microsphere (diameter *D*: 65 μm)	1064 nm ns-pulsed laser, room temperature (RT), free space coupling	480	/	20 mW	/	/

**1999** [8]	Er^{3+}-doped ZBLAN microsphere (*D*: 120 μm)	801 nm CW laser, RT, prism coupling	539	/	30 μW	/	/
**2009** [9]	Er^{3+}-doped silica microtoroid	1450 nm CW laser, RT, tapered optical fiber coupling	550	/	690 μW	/	/
**2010** [10]	Er^{3+}-doped ZBNA microsphere (*D*: 60 μm)	978 nm LD laser, RT, tapered optical fiber coupling	550	<1 nm	3 μW	~1000	/
**2013** [11]	Tm^{3+}-doped silica microtoroid	1064 nm CW laser, RT, tapered optical fiber coupling	450	/	32 μW	/	/
**2013** [12]	$NaYF_4$:Yb^{3+}/Er^{3+}@$NaYF_4$ nanoparticle-coated micro bottle (*D*: 80 μm)	980 nm ns-pulsed laser, RT, free space coupling	407	< 0.2	8.5 kW/cm^2	>2035	Note
			540		5.5 kW/cm^2	>2700	
			655		7.5 kW/cm^2	>3275	
**2017** [13]	Yb^{3+}/ Tm^{3+}/Er^{3+} doped $NaYF_4$ hexagonal microrod (radius *R*: 4 μm)	980 nm ns-pulsed laser, RT, free space coupling	450	/	4.8 mJ/cm^2	/	/
			540	/	3.8 mJ/cm^2	/	
			654	/	3.0 mJ/cm^2	/	
**2018** [14]	Yb^{3+}/Er^{3+} doped fluorosilicate glass microsphere (*D*: 58 μm)	980 nm LD, RT, tapered optical fiber coupling	545	/	52.5 μW	/	/
**2018** [15]	$NaYF_4$:Gd^{3+}/Tm^{3+}@$NaGdF_4$ nanoparticle-coated polystyrene microsphere (*D*: 5 μm)	1064 nm CW laser, RT, free space coupling	450	0.7	44 kW/cm^2	643	/
**2021** [16]	$NaGdF_4$:Yb^{3+}/Tm^{3+}(or Er^{3+})@$NaGdF_4$ nanoparticle-coated polystyrene microsphere (*D*: 5 μm)	980 nm CW laser, RT, free space coupling	471	0.25	4 W/cm^2	1884	/
			557	0.5	40 W/cm^2	1114	
			665	0.5	40 W/cm^2	1330	
**2021** [17]	$NaYF_4$:Yb^{3+}/Er^{3+}(or Tm^{3+}) embedded amorphous microspheres (*D* < 3 μm)	980 nm CW laser, RT, free space coupling	474	0.48	1.5 kW/cm^2	988	/
			525	0.27	817 W/cm^2	1944	
			550	0.28	589 W/cm^2	1964	
			660	0.48	4.7 W/cm^2	1375	
**2022** [18]	Yb^{3+}-Tm^{3+}-Er^{3+} co-doped silica microsphere (*D*: 53 μm)	975 nm LD laser, RT, tapered optical fiber coupling	480	/	300 μW	/	/
			550	/	500 μW	/	/
			660	/	90 μW	/	/
**2022** [19]	Yb^{3+}-Er^{3+} co-doped silica microsphere (*D*: 57 μm)	975 nm LD laser, RT, tapered optical fiber coupling	410		170 μW		
			450		2.7 μW		
			560		18 μW		
			660		0.18 μW		
**This work**	**Yb^{3+}/Er^{3+}, or Yb^{3+}/Tm^{3+} doped nano-glass composite microspheres (*D* ~ 30 μm)**	**978 nm LD laser, RT, tapered optical fiber coupling**	**470**	**0.204**	**183 μW**	**2304**	**0.21%**
			**545**	**0.164**	**150 μW**	**3323**	**0.24%**
			**650**	**0.156**	**157 μW**	**4167**	**0.26%**

Note: the original description in the main text is inconsistent with Fig. 5(b), and the correct values were given here

References

[1] Kang, S. et al. Enhanced 2 μm mid-infrared laser output from Tm^{3+}-activated glass ceramic microcavities, *Laser Photonics Rev.* **14**, 1900396 (2020).

[2] Shannon, R., Shannon, R., Medenbach, O. & Fischer, R. Refractive index and dispersion of fluorides and oxides, *J. Phys. Chem. Ref. Data* **31**, 931-970 (2002).

[3] Yang, S., Wang, Y. & Sun, H. Advances and prospects for whispering gallery mode microcavities, *Adv. Opt. Mater.* **3**, 1136-1162 (2015).

[4] Thung, Y. et al. Ultrahigh quality microlasers from controlled self-assembly of ultrathin colloidal semiconductor quantum wells, *Laser Photonics Rev.* **17**, 2200849 (2023).

[5] Judd, B. Optical absorption intensities of rare-earth ions, *Phys. Rev.* **127**, 750-61 (1962).

[6] Ofelt, G. Intensities of crystal spectra of rare-earth ions, *J. Chem. Phys.* **37**, 511-20 (1962).

[7] Fujiwara, H. & Sasaki, K. Upconversion lasing of a thulium-ion-doped fluorozirconate glass microsphere, *J. Appl. Phys.* **86**, 2385-2388 (1999).

[8] Klitzing, W. et al. Very low threshold lasing in Er^{3+} doped ZBLAN microsphere, *Electron. Lett.* **35**, 1745-1746 (1999).

[9] Lu, T., Yang, L., Loon, R., Polman, A. & Vahala, K. On-chip green silica upconversion microlaser, *Opt. Lett.* **34**, 482-484 (2009).

[10] Wu, Y., Ward, J. & Chormaic, S. Ultralow threshold green lasing and optical bistability in ZBNA (ZrF_4-BaF_2-NaF-AlF_3) microspheres, *J. Appl. Phys.* **107**, 033103 (2010).

[11] Mehrabani, S. & Armani, A. Blue upconversion laser based on thulium-doped silica microcavity, *Opt. Lett.* **38**, 4346-4348 (2013).

[12] H. Zhu, X. Chen, L. M. Jin, Q. J. Wang, F. Wang, and S. F. Yu, Amplified Spontaneous Emission and Lasing from Lanthanide-Doped Up-Conversion Nanocrystals, *ACS Nano* 7, 11420-11426 (2013)

[13] Ting Wang, Huan Yu, Chun Kit Siu, Jianbei Qiu, Xuhui Xu, and Siu Fung Yu, White-Light Whispering-Gallery-Mode Lasing from Lanthanide Doped Upconversion NaYF4 Hexagonal Microrods, *ACS Photonics* 4, 1539-1543 (2017)

[14] Wang, X. et al. Single mode green lasing and multicolor luminescent emission from an Er^{3+}-Yb^{3+} co-doped compound fluorosilicate glass microsphere resonator, *OSA Continuum* **1**, 261-273 (2018).

[15] Bravo, A. et al. Continuous-wave upconverting nanoparticle microlasers, *Nat. Nanotech.* **13**, 572-577 (2018).

[16] Yang, X., Lyu, Z., Dong, H., Sun, L. & Yan, C. Lanthanide Upconverted Microlasing: Microlasing Spanning Full Visible Spectrum to Near-Infrared under Low Power, CW Pumping, *Small* **17**, 2103140 (2021).

[17] B. S. Moon, T. K. Lee, W. C. Jeon, S. K. Kwak, Y. J. Kim, D. H. Kim, Continuous-wave upconversion lasing with a sub-10 W cm^{-2} threshold enabled by atomic disorder in the host matrix, *Nat. Commun.* 12, 4437 (2021).
[18] Jiang, B. et al. Room-temperature Continuous-Wave Upconversion White Microlaser Using a Rare-earth-Doped Microcavity, *ACS Photonics* **9**, 2956-2962 (2022).
[19] B. Jiang, S. Zhu, L. Ren, L. Shi, and X. Zhang, Simultaneous ultraviolet, visible, and near-infrared continuous-wave lasing in a rare-earth-doped microcavity, Adv. Photon. 4, 046003 (2022).
